# A Ubiquitous Volatile in Noctuid Larval Frass Attracts a Parasitoid Species

**DOI:** 10.3390/biology14081007

**Published:** 2025-08-06

**Authors:** Chaowei Wang, Xingzhou Liu, Sylvestre T. O. Kelehoun, Kai Dong, Yueying Wang, Maozhu Yin, Jinbu Li, Yu Gao, Hao Xu

**Affiliations:** 1 State Key Laboratory of Agricultural and Forestry Biosecurity, College of Plant Protection, Nanjing Agricultural University, Nanjing 210095, China; 18815575879@163.com (C.W.); 2023102292@stu.njau.edu.cn (S.T.O.K.); 2023802253@stu.njau.edu.cn (K.D.); 2 Key Laboratory of Soybean Disease and Pest Control, Ministry of Agriculture and Rural Affairs, Nanjing Agricultural University, Nanjing 210095, China; 3 Institute of Plant Protection, Suzhou Academy of Agricultural Sciences, Suzhou 234000, China; lxz8610@163.com (X.L.); wyysznky@163.com (Y.W.); 2018102025@njau.edu.cn (M.Y.); lijinbu0991@sina.com (J.L.); 4 Department of Agriculture, Suzhou Vocational and Technical College, Suzhou 234000, China; 5 College of Plant Protection, Jilin Agricultural University, Changchun 130118, China

**Keywords:** tritrophic interaction, parasitic wasp, foraging behavior, plant volatile, biological control, pest management

## Abstract

Natural enemies commonly probe larval bodies and frass with their antennae for prey hunting. However, the attractants to natural enemies emitted directly from hosts and host-associated tissues remained largely unknown. Here, we used two generalist noctuid species, *Helicoverpa armigera* (Hübner) and *Spodoptera frugiperda* (JE Smith), along with the larval endoparasitoid *Microplitis mediator* (Haliday) to address the question. Extracts of larval frass of both the noctuid species were strongly attractive to *M. mediator* females when hosts were fed either maize, cotton, soybean leaves, or an artificial diet without leaf tissues. By using a combination of electrophysiological measurements and behavioral tests, we found that the attractiveness of frass mainly relied on a volatile compound ethyl palmitate. The compound was likely to be a by-product of host digestion involving gut bacteria because an antibiotic supplement in diets reduced the production of the compound in frass and led to the decreased attractiveness of frass to the parasitoids. In contrast, extracts of the larval bodies of both the noctuid species appeared to be less attractive to the parasitoids than their respective fecal extracts, independently of types of food supplied to the larvae. Altogether, larval frass of the two noctuid species was likely to be more important than their bodies in attracting the endoparasitoid species, and the main attractant of frass was probably one of the common metabolites of digestion involving gut microbes, and its emission is likely to be independent of host plant species.

## Simple Summary

It is increasingly becoming common knowledge that predators and parasitoids use herbivore-induced plant volatiles (HIPVs) to locate their herbivore hosts. However, many natural enemies are also attracted to odors emitted directly from hosts or host-associated tissues. Little is known about the mechanism of the latter phenomenon. Here, we found that the larval bodies of two noctuid species had similar chemical compositions, as did their larval frass, which were also largely independent of the types of food (maize leaves versus an artificial diet without leaf tissues) supplied to the larvae. The volatile compound ethyl palmitate of frass was strongly attractive to the larval endoparasitoid *Microplitis mediator* (Haliday). However, the compound was rarely found in extracts of the larval bodies, which explains why the larval frass was more attractive to the parasitoids than their respective larval bodies. We also hypothesize that ethyl palmitate and some other compounds were common metabolites of gut digestion involving bacteria based on comparable analyses of samples obtained from the larvae fed diets supplemented with an antibiotic compound.

## 1. Introduction

Parasitoids are important agents in biological control programs against insect pests. They mainly rely on olfactory cues to locate their hosts in a complex chemical environment. Plant volatiles are released in large amounts induced by herbivory and serve as an important cue for parasitoids locating hosts [1]. However, herbivore-induced plant volatiles (HIPVs) are sometimes not host-specific (e.g., green leaf volatiles) [2,3,4], and host or host-associated cues are important for confirming the presence of hosts [5,6,7].

Odors of host by-products, such as frass, silks, and cocoons, often act as kairomones (benefiting natural enemies, but harmful to the hosts in this case) to attract parasitoids [8,9,10,11,12,13,14]. Since insect feces appear to be a reliable cue for many natural enemies to find prey/hosts, shelter-dwelling larvae of several lepidopteran families have evolved to perform an unusual behavior to avoid attacks by ejecting fecal pellets away from their feeding sites [15,16]. In addition, the smells of hosts are also attractive to parasitoids. For example, parasitoids often eavesdrop on host pheromones emitted by adults [5,17,18,19], and some larval parasitoids are directly attracted to host larval smells [20,21,22,23,24]. In the circumstances where HIPVs are not available, host and host-associated smells at the immature stage might be the principal cue for attracting parasitoid species, including those parasitizing pollinator hosts [9,25], belowground insects [20,26], and storage pests [27,28].

The attractiveness of host frass to parasitoids has often been found to be independent of the types of food consumed by polyphagous hosts, and both feces obtained from leaves and non-leaf contained artificial diets are attractive to parasitoid species [11,29,30]. Frass of hosts that are fed non-leaf plant tissues (e.g., roots, grains, and pollens) is also attractive to parasitoids [25,26,27]. These findings suggest that the attractants in feces are possibly common metabolites of host digestion, independently of types of host food. However, several studies have also revealed that ingested leaf tissues in host bodies and feces played an additional role in attracting natural enemies [19,31,32,33,34]. Even more, the attractiveness of frass is sometimes entirely dependent on the presence of the ingested leaf tissues, and frass obtained from non-leaf contained artificial diets is not attractive to parasitoids [31]. Therefore, identifying the attractants of frass is necessary for gaining a better understanding on how natural enemies use fecal cues for prey finding.

Recently, the roles of gut bacteria of hosts have received increasing attention in terms of mediating foraging behaviors of natural enemies. For example, gut bacteria of the Colorado potato beetle *Leptinotarsa decemlineata* Say produce defense compounds (e.g., mandelonitrile) to ward off natural enemies [35]. Gut bacteria are also responsible for producing some volatile compounds that mediate intraspecific aggregations in the German cockroach *Blattella germanica* (L.) [36]. When those microbe-related cues are reliable, it would be expected that parasitoid species might use them for locating hosts, as suggested in previous papers [37,38,39,40]. However, the specific compounds that are released because of activities of insect symbionts, but betray hosts, remain largely unknown.

The cotton bollworm *Helicoverpa armigera* (Hübner) (Lepidoptera: Noctuidae) and the fall armyworm *Spodoptera frugiperda* (JE Smith) (Lepidoptera: Noctuidae) are of great importance in agriculture. *H. armigera* occurs in many regions, including Europe, Africa, and Asia, and damages plant species of 68 families [41]. *S. frugiperda*, native to the New World, is now a devastating invasive pest of maize in Africa and Asia [42,43], and it also feeds on other crops, including rice, peanuts, and sorghum [44,45]. The solitary larval endoparasitoid *Microplitis mediator* (Haliday) (Hymenoptera: Braconidae) is widely distributed in Europe and Asia, and parasitizes a variety of noctuid species, including *H. armigera* and *S. frugiperda* [46,47,48]. This species is strongly attracted by HIPVs [46,49]. However, little is known about how the parasitoid species uses host/host-associated cues for locating hosts.

In this study, we first evaluated the relative attraction of the parasitoids to extracts of larval bodies and frass of the two noctuid species, taking into account the effects of food types supplied to the host larvae. By using a combination of chemical analyses and electroantennographic recordings, we identified a key compound that was responsible for the attractiveness to the parasitoids. Finally, we studied the relationship between the production of the compound and gut microbes by using an antibiotic supplement in diets.

## 2. Materials and Methods

Insects and Plants—The parasitoid species *M. mediator* first emerged from *H. armigera* caterpillars collected from soybeans grown in fields in Suzhou (33.636° N, 117.082° E), Anhui Province, China. When rearing, five female parasitoids were used to parasitize about 50 caterpillars of *H. armigera* (about 5 d old) that were kept in square plastic boxes (15 × 13 × 5 cm) and fed a wheat germ-based artificial diet (see below). Parasitism lasted for 24 h in a 25 °C incubator (LD 16:8 h), with honey provided as food for the female parasitoids, as described in a previous publication [50]. Wasp cocoons were collected from the plastic boxes about 2 weeks after parasitism and transferred to 30 × 30 × 30 cm nylon rearing tents (Hongrixing, Xiamen, China), and then provided with honey and moist cotton wool in a 25 °C incubator (LD 16:8 h). We used the naïve female wasps (2–7 d old after emergence) for olfactometer bioassays as described below.

The rearing of the host species *H. armigera* and *S. frugiperda* was initiated using caterpillars collected from soybean and maize plants, respectively, in Suzhou (33.636° N, 117.082° E), Anhui Province, China. Caterpillars of both these species were reared on a modified form of the wheat germ-based artificial diet as described in a previous paper [51]. Specifically, antimicrobial agents, including methyl paraben, sorbic acid, and chlortetracycline, were removed from the diet. Rearing was first carried out in the same plastic boxes used for parasitism, with 50 caterpillars placed in each box. The larvae were reared in groups before the fourth instar, and then individually in plastic tubes (3 × 5 cm) until pupation. The pupae of each species were transferred to the same type of rearing tent (30 × 30 × 30 cm) used to keep the wasps, and we provided emerged moths with 10% honey solution as food.

Seedlings of maize, cotton, and soybean were planted in plastic tubes (OD = 4.5 cm, L = 13 cm) and grown for three weeks in an incubator (28 °C, LD = 16:8 h). The leaves were harvested daily to feed caterpillars of both the species, starting with 50 individuals (4-d old) in the square plastic boxes. Thus, caterpillars of the both species were fed either maize, cotton, soybean leaves, or the artificial diet. After 6 days of feeding, frass and caterpillars were collected and used for odor extraction (see below).

Sample collections on diets supplemented with the antibiotic—Two micrograms of tetracycline (98%, Macklin Ltd., Shanghai, China) were added to one liter of the artificial diet when the temperature of the liquid diet had cooled to 55 °C during diet preparation and the amount of the antibiotic compound was similar to that of other antimicrobial agents of the artificial diet described by Merkx-Jacques and Bede [51]. In addition, 20 µg of tetracycline that was dissolved in 1 mL of water was evenly sprayed on leaves of maize seedlings (2–3 weeks old), and the control maize seedlings were applied 1 mL of water, similar to a previous paper [37]. Fifty caterpillars (about 5 d old after hatching) were weighed, and then we fed them with one of the two types of food (maize leaves or the artificial diet) supplemented either with or without the antibiotic compound in the square plastic boxes (15 × 13 × 5 cm). The frass and caterpillars were collected twice from the boxes after 3 d or 6 d of feeding for odor extraction (see below). The caterpillars were weighed again in groups of ten individuals, and their weight gain rates were calculated. The data were analyzed with the *t*-test performed by SigmaPlot 12.5.

Solvent extraction—After the two periods (the first or the second 3 d periods, i.e., 1st–3rd and 4th–6th d) of feeding, the larvae that were randomly chosen from the rearing boxes were killed by placing them in a freezer at −20 °C for 10 min. Compounds in 100 mg of caterpillars (about 10 larvae after 6 d of feeding and 20 larvae after 3 d of feeding) and in 100 mg of their frass were extracted, respectively, by adding 500 µL of dichloromethane (DCM) (*n* = 5). During extraction, the samples were vibrated for about 30 s on a Vortex-Genie 2 mixer (Scientific Industries, Bohemia, NY, USA). The liquid supernatant was removed with a syringe and stored in vials at −20 °C before performing bioassays or chemical analyses.

Bioassays—A four-arm olfactometer was used to test the attractiveness of frass and caterpillar extracts to the parasitic wasps as previously described [52]. Each olfactometer arm delivered a gentle stream of air (0.6 L/min) into the central release chamber. The naïve female wasps were released in groups of six into the olfactometer, where they were allowed to choose among the treatments for up to 30 min. At the end of this period, or as soon as all six wasps had made a choice, the result was recorded. Then, the tested wasps were removed, and a new group of six naïve wasps was released into the olfactometer. Each replicate included four releases of six wasps. Each experiment consisted of six replicates (144 wasps in total, *n* = 6). Glassware was cleaned using the method described by Desurmont et al. [53], and the positions of the treatments in the olfactometer were changed randomly for each replicate.

Samples (10 μL) were applied on paper strips (1 × 3 cm) that had been placed on two opposite arms of the four-arm olfactometer, and the rest arms in between were treated with the same volume of control solvent (DCM) likewise. The design aimed to minimize the interactions between the tested parasitoids in the olfactometers [54]. Before testing, the paper strips were placed into a running fume hood for 15 min to allow the solvent to evaporate [55].

The data obtained from the olfactometer test did not meet the normality assumptions. Therefore, a Poisson generalized linear mixed model (GLMM) was run to assess the effects of the treatments (fixed factor) on the insect choices, with replicates of the experiment as a random blocking factor [56], which was performed in R version 3.0.2 with the package lme4 [57].

Gas Chromatography–Mass Spectrometry Analyses—The samples were analyzed with an Agilent 8890 gas chromatograph (GC), coupled with an Agilent 5977B mass spectrometer (MS) detector (Agilent Technologies Inc., Palo Alto, CA, USA). For each sample, 2 μL of extract was injected into the GC column using 10% split mode. The temperature of injector was 250 °C. Helium was used as the carrier gas within a non-polar column (HP-5ms, 30 m, 0.25 mm ID, 0.25 μm film thickness, Agilent Technologies) at a constant flow rate (1 mL/min). The oven temperature was initially set to 40 °C, held for 7 min, and then increased at a rate of 5 °C per minute to 55 °C, followed by a ramp of 15 °C per minute to 250 °C, and then held at this temperature for 2 min. The compounds were first identified by mass spectrometry analysis by comparing their mass spectra with the NIST mass spectral library database (Version 2.4). Most compounds were then confirmed by injection of commercially available standards, including (2*S*,3*S*)-(+)-butanediol (98%, Bidepharm Ltd., Shanghai, China), meso (*R*,*S*)-2,3-butanediol (99%, Macklin Ltd., Shanghai, China), pentadecane (99%, Macklin, Shanghai), palmitoleic acid (98%, Macklin, Shanghai), palmitic acid (98%, Mreda, Beijing, China), benzaldehyde (98%, Macklin, Shanghai), and ethyl palmitate (98%, Macklin, Shanghai). However, the identifications of 2-ethylhexanol and (*E*)-coniferol were considered as tentative based on suggestions by the NIST database.

Two internal standards n-octane and nonyl acetate (200 ng each combined in 10 µL dichloromethane) were added to each sample as references and were used to quantify the collected compounds. Pairwise comparisons of the compound amounts of extracts were carried out with the *t*-test performed using SigmaPlot 12.5.

Gas Chromatography–Electroantennographic Detection (GC-EAD) Analyses—The GC-EAD system consisted of an Agilent 6890 GC, equipped with a flame ionization detector (FID) and a non-polar column (HP-5ms, 30 m, 0.25 mm ID, 0.25 μm film thickness, Agilent Technologies), and was coupled to an EAD setup (Syntech, Hilversum, The Netherlands). Aliquots (2 µL) of the samples were injected splitless into the GC injector (280 °C) at an initial column temperature of 40 °C, and then the temperature was increased at a rate of 15 °C per minute to 250 °C. The GC effluent was split (split ratio FID:EAD = 1:3) by using a µFlow splitter (Gerstel, Mühlheim, Germany), and 25 mL/min of make-up gas (nitrogen) was added to carry the separated compounds through two deactivated capillaries: one leading to the FID, and the other leading to the EAD setup. The EAD outlet entered into purified and humidified constant airflow that was directed over the antennal preparation.

To prepare the antennae, *M. mediator* females were anesthetized with CO_2_, and their heads were pulled out from the body with forceps, and the two tips of both antennae were cut and inserted into a glass capillary [58]. To close the electric circuit, the severed head was connected via the neck to another glass capillary. The capillaries were filled with insect Ringer solution (5 g NaCl; 0.42 g KCl; 0.19 g CaCl_2_ in 1000 mL demineralized water). Each EAD test was replicated five times with different insect heads.

## 3. Results

**Figure 1:**
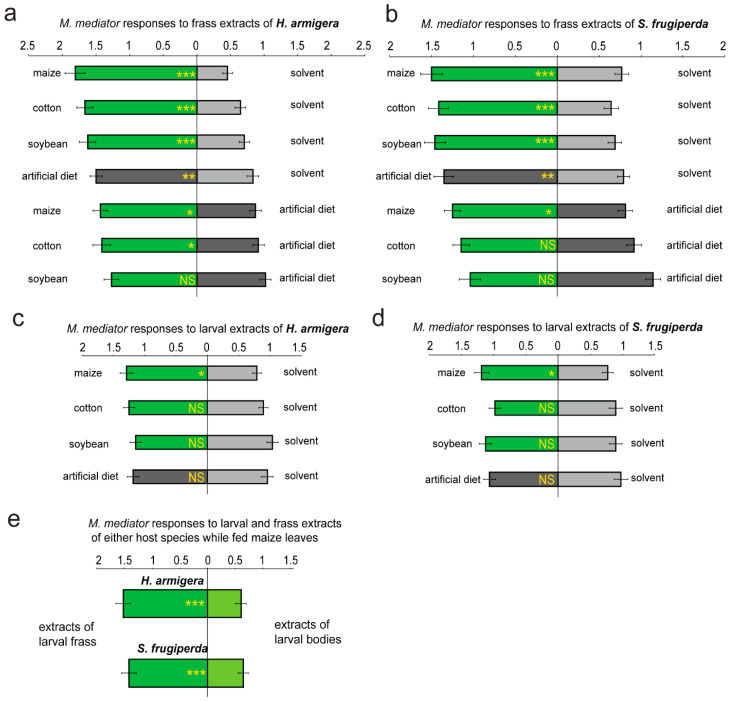
Attractiveness of solvent extracts of the larval bodies and frass of the two noctuid species to the parasitoid *M. mediator*. The response of *M. mediator* to odors of the larval frass of *H. armigera* (**a**) or *S. frugiperda* (**b**) when their larvae were fed either maize, cotton, soybean leaves, or an artificial diet without leaf tissues. The same volume (10 μL) of dichloromethane was used as the solvent control. Similarly, the response of *M. mediator* to odors of the larval bodies of *H. armigera* (**c**) or *S. frugiperda* (**d**) was also tested when the larvae were fed various types of food. (**e**) The relative attractiveness to *M. mediator* of the larval frass and bodies was compared when the larvae of either species were fed maize leaves. The asterisks on the bar graphs indicate significant differences between the treatments (GLMM, *n* = 6; *: *p* as 0.01~0.05, **: *p* as 0.001~0.01, ***: *p* < 0.001). “NS” indicates no significant difference between the treatments.

The *M. mediator* females were more strongly attracted to the larval frass of both the noctuid hosts compared to the solvent controls when the caterpillars were fed either maize, cotton, soybean leaves (GLMM, *p* < 0.001, *n* = 6), or the artificial diet (*p* < 0.01) (Figure 1a,b). In both the species, the frass obtained from the larvae fed maize leaves was more attractive to the parasitoids than the frass obtained from the larvae fed the artificial diet (GLMM, *p* < 0.05, *n* = 6) (Figure 1a,b). Similarly, frass obtained from the *H. armigera* larvae fed the cotton leaves was more attractive to the parasitoids than that obtained from larvae fed the artificial diet (Figure 1a). However, the frass derived from soybean leaves was as attractive as that derived from the artificial diet, regardless of the species (GLMM, *p* > 0.05) (Figure 1a,b).

Similar tests on extracts of the larval bodies indicated that the body odors of both the host species were not significantly attractive to the parasitoids when the larvae were fed either cotton, soybean leaves, or the artificial diet (GLMM, *p* > 0.05, *n* = 6) (Figure 1c,d). However, when the larvae of both the species were fed maize leaves, the attractiveness of their body extracts to the parasitoids was significantly increased as opposed to the solvent control (GLMM, *p* < 0.05) (Figure 1c,d).

To obtain a clearer vision of the relative importance of the larval frass and bodies in attraction of the parasitoids, we also compared the attractiveness of the larval bodies and frass to the parasitoids when the larvae were fed maize leaves, and found that the attractiveness of frass was significantly stronger, independently of the host species (GLMM, *p* < 0.001, *n* = 6) (Figure 1e). The comparable tests were not performed on the samples derived from the other types of food (including soybean, cotton leaves, and the artificial diet) because the attractiveness of the larval bodies given these diets was not significantly stronger than that of the solvent control (GLMM, *p* > 0.05, *n* = 6) (Figure 1c,d). 

**Figure 2:**
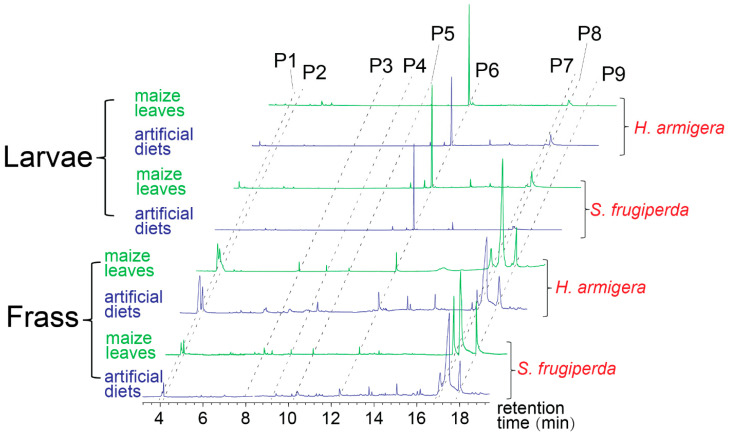
Typical chromatographs of the solvent extracts of larval bodies or frass of both the noctuid species when the larvae were fed either maize leaves or the non-leaf contained artificial diet. P1 and P2: two stereoisomers of 2, 3-butanediol; P3: benzaldehyde; P4: 2-ethylhexanol; P5: (*E*)-coniferol; P6: pentadecane; P7: palmitoleic acid; P8: palmitic acid; P9: ethyl palmitate. The amounts of these compounds are presented in Table 1. The two 2,3-butanediol stereoisomers are typical VOCs of bacteria [59], and benzaldehyde, 2-ethylhexanol, and (*E*)-coniferol are considered as leaf-associated volatiles in this paper because they were only found in the frass obtained from the larvae fed maize leaves, but not in frass of those fed the artificial diet.

The chemical compositions of larvae and frass were different. For example, the alkane compound pentadecane was the main compound of caterpillars of both the noctuid species, but it was released in much lower amounts in fecal extracts of both the species (*t*-test, *p* < 0.05, *n* = 5) (Figure 2 and Table 1). In contrast, two 2,3-butanediol stereoisomers, two fatty acids (palmitoleic acid and palmitic acid), and the ester compound ethyl palmitate were emitted in higher amounts by the frass of both the species than by the larval bodies (*t*-test, *p* < 0.05) (Figure 2 and Table 1). The diet types also led to different amounts of compounds produced by the frass of both the species. Three compounds, i.e., benzaldehyde, 2-ethylhexanol, and (*E*)-coniferol, were exclusively found in the frass obtained from the larvae fed maize leaves, but not in the frass of those fed the artificial diet (Figure 2 and Table 1; they are referred to as leaf-associated volatiles in this paper). However, amounts of the other compounds of the larval frass and bodies were independent of the diet types in both the noctuid species (Table 1, *t*-test, *p* > 0.05, *n* = 5).

**Figure 3:**
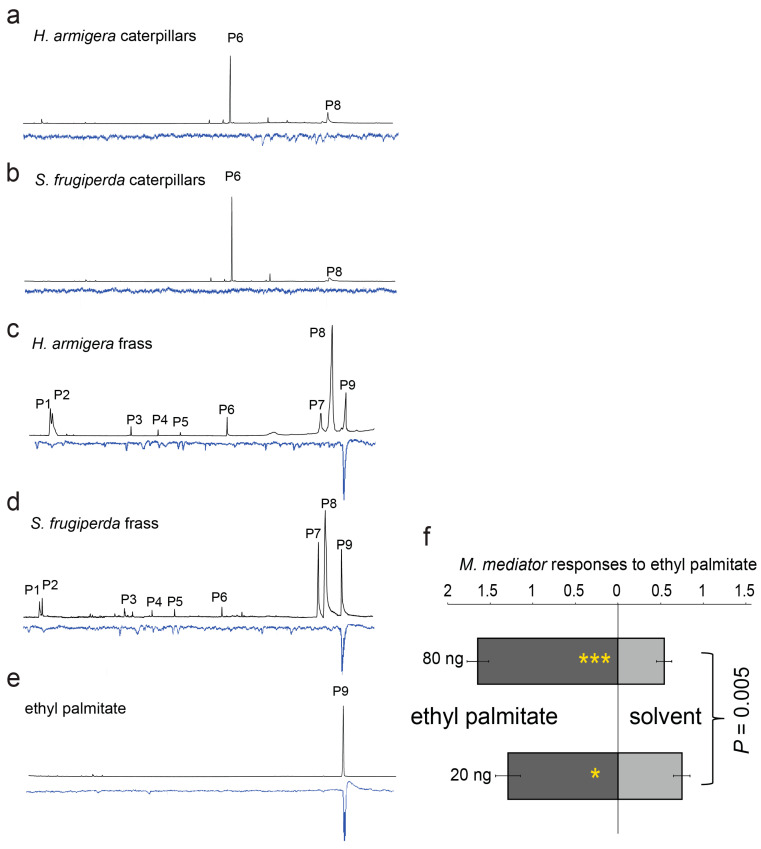
Electrophysiological responses of the antennae of the *M. mediator* females to solvent extracts of the *H. armigera* or *S. frugiperda* caterpillars (**a**,**b**) or frass (**c**,**d**) when their larvae were fed maize leaves. In addition, the electrophysiological response to the synthetic standard ethyl palmitate was also tested (**e**). Attractiveness of ethyl palmitate to the parasitoid *M. mediator* females at various doses was tested, where the same volume (10 μL) of dichloromethane was used as the solvent control (**f**) (GLMM with Tukey’s post-hoc test, *n* = 6; *: *p* as 0.01~0.05, ***: *p* < 0.001).

The GC-EAG analyses showed that ethyl palmitate in the fecal extracts of both the species stimulated a strong antennal response in the parasitoids (Figure 3c,d), whereas the extracts of larval bodies did not elicit apparent EAG responses (Figure 3a,b). The commercial standard of ethyl palmitate elicited a similar antennal response (Figure 3e). The behavioral tests indicated that the compound was strongly attractive to the parasitoids (Figure 3f; GLMM, *: *p* as 0.01~0.05, ***: *p* < 0.001, *n* = 6), with a positive change in relation to the amounts of compound applied (GLMM with Tukey’s post-hoc test, dose effect: F_1,188_ = 8.2, *p* = 0.005).

**Figure 4:**
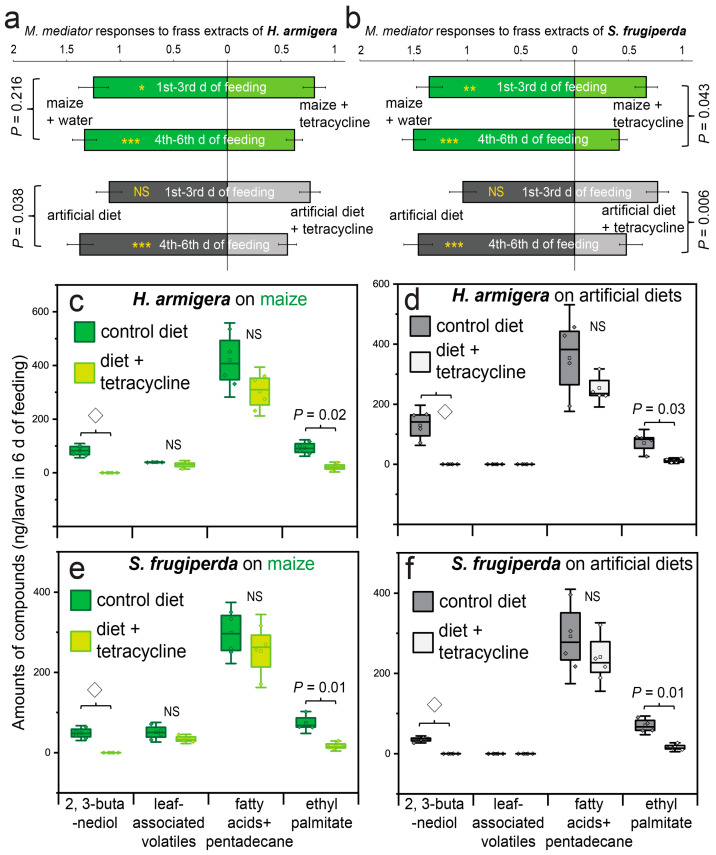
Attractiveness of the solvent extracts of larval frass of the two noctuid species (**a**,**b**) to the parasitoid *M. mediator* when the larvae were fed maize leaves or the artificial diet supplemented with the antibiotic compound tetracycline. The feeding of larvae lasted either 3 or 6 days (i.e., 1st~3rd or 4th~6th days of feeding). The asterisks on the bar graphs indicate significant differences between the treatments (GLMM with Tukey’s post-hoc test, *n* = 6; *: *p* as 0.01~0.05, **: *p* as 0.001~0.01, ***: *p* < 0.001). “NS” indicates no significant difference between the treatments. The amounts of compounds found in larval frass of the two noctuid species (**c**,**d** for *H. armigera*; **e**,**f** for *S. frugiperda*) when they were fed one of two types of food (the maize leaves shown in **c**,**e**; the artificial diet shown in **d**,**f** ) that had been (or not been) supplemented with tetracycline for 6 days. The differences were analyzed with the *t*-test (*n* = 4). “◇” is used to highlight that the compounds were not detected in the frass obtained from the larvae fed diets supplemented with tetracycline.

The frass collected from the caterpillars fed maize leaves supplemented with tetracycline was significantly less attractive to the parasitoids after both the early (first–third days) and the late (fourth–sixth days) feeding periods compared to the frass from the larvae fed control maize leaves (Figure 4a,b; GLMM, *: *p* as 0.01~0.05, **: *p* as 0.001~0.01, ***: *p* < 0.001, *n* = 6). Similar results were also obtained when tetracycline was added to the artificial diet, although the reduction in attractiveness became significant only after 6 days of feeding in both the species (Figure 4a,b), suggesting that the longer feeding duration (6 d) on the tetracycline-treated diets led to a stronger reduction in attractiveness to the parasitoids (Figure 4a,b; GLMM with Tukey’s post-hoc test, effects of feeding days: F_1,188_ = 4.1~7.4, *p* < 0.05; however, no such difference appeared in *H. armigera* when the larvae were fed maize leaves: F_1,188_ = 1.5, *p* = 0.216).

Additions of tetracycline to maize leaves or the artificial diet ceased the production of the two 2,3-butanediol stereoisomers in frass of both the species after 6 days of feeding (Figure 4c–f). Meanwhile, the production of ethyl palmitate was also reduced (*t*-test, *p* < 0.05, *n* = 5). However, the amounts of the three leaf-associated volatiles (i.e., benzaldehyde, 2-ethylhexanol, and (*E*)-coniferol), pentadecane, and the two fatty acids were not significantly affected by dietary addition of tetracycline in both types of food (*t*-test, *p* > 0.05, *n* = 5).

**Figure 5:**
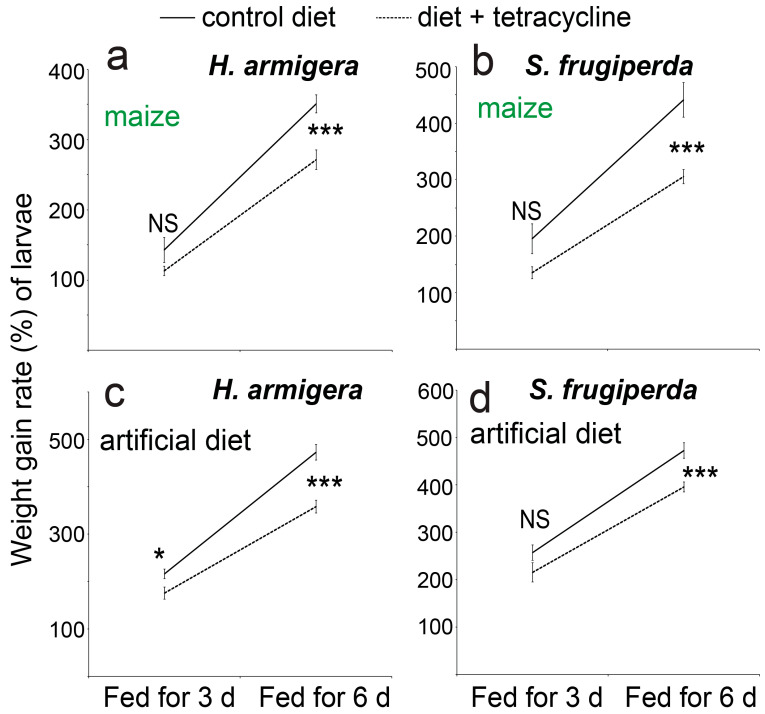
The weight gain rates (%) of caterpillars of the two noctuid species when the two types of food (maize leaves shown in **a**,**b** and the artificial diet shown in **c**,**d**) were supplemented with the antibiotic compound tetracycline. Feeding lasted 3 or 6 days. The differences were analyzed with the *t*-test (*n* = 5, *: *p* as 0.01~0.05, ***: *p* < 0.001). “NS” indicates no significant difference between the treatments.

Providing the artificial diet or maize leaves that had been supplemented with tetracycline led to lower weight gain rates of caterpillars of both the species, and the differences were generally statistically significant after 6 d of feeding (*t*-test, *p* < 0.001, *n* = 5) (Figure 5).

## 4. Discussion

Parasitoid species rely on various odor cues to locate their host species, which include HIPVs, host body odors, and host-associated smells, such as those emitted from silks and frass [1,9]. Host fecal odors have often been reported as a reliable cue for natural enemies to locate their hosts or prey [10,11,12,13,14,15,16]. Unlike eavesdropping on host pheromones in a relatively long range [5,18], natural enemies normally sense host fecal odors in a relatively short range. For example, using antennae to probe frass (compounds) is a common behavior of parasitoid species in confirming the presence of host species [11,60]. Although such interactions between hosts and parasitoids are common, the underlying mechanisms remain largely unexplored. By combining behavioral bioassays, chemical analyses, and electrophysiological analyses, we found that the ester compound ethyl palmitate was responsible for the attractiveness of larval frass of the two noctuid species to the parasitoid *M. mediator*.

Relative importance of smells of host frass and bodies in attracting parasitoids—Many studies have found that odors of host bodies and/or frass are attractive to parasitoid species [20,21,34,61]. However, the relative importance of these two kinds of odor in attracting parasitoids has rarely been compared and discussed. Shelter-dwelling larvae of several lepidopteran families have evolved to perform an unusual behavior to avoid attacks from natural enemies by ejecting fecal pellets away from their homes [15,16], suggesting that larval frass of some lepidopterans is important in attracting natural enemies, as also confirmed in the present study. We also found that the odors of larval bodies were not significantly attractive to the parasitoids in general, although the presence of leaf tissues (e.g., maize) in larval bodies sometimes increased attractiveness to the parasitoids. Therefore, this study concluded that larval frass of the two noctuid species was more important than host bodies in attracting the parasitoids. This conclusion is in line with some previous studies that also found frass to be more attractive to natural enemies than host bodies [20,26,30].

However, some parasitoid species are not significantly attracted to their host’s frass [22,62], and some other signals, such as visual cues and vibrations, possibly work in confirming the presence of host species by parasitoids [62,63]. Thus, the success of host foraging may depend on various cues, with variations according to parasitoid species [64].

The underlying mechanism of why fecal attractiveness to parasitoids does not depend on food types—The frass obtained from larvae fed various types of food, including maize, cotton, soybean leaves, and the artificial diet, was strongly attractive to the parasitoid *M. mediator*, suggesting that the attractiveness was largely independent of the larval diets. Similar results have also been observed in interactions between other generalist herbivores and their parasitoids [11,30]. However, the presence of ingested leaf tissues (e.g., maize) in larval bodies and frass increased the level of attractiveness to *M. mediator*, highlighting that leaf residues of some plant species in larval bodies and frass indeed play an additional role in attraction of natural enemies, as previously suggested [19,31,32].

The innate attractiveness of larval frass to parasitoids even occurs in non-host species. For example, the braconid parasitoid *Cotesia rubecula* is attracted by frass of the non-host species *Pieris napi,* and the level of attractiveness was as strong as that of the host species *Pieris rapae* [10]. These results indicate that the attractiveness of frass to *C. rubecula* relied on some conserved compounds that remained unchanged in the phylogenetically related species.

The chemical compositions of larval frass that was obtained from the insects fed one of the two types of diet could reveal the underlying mechanism. Although leaf-associated volatiles (i.e., benzaldehyde, 2-ethylhexanol, and (*E*)-coniferol) were only found in the frass obtained from the larvae fed maize leaves, but not in the frass from those given the artificial diet, the other compounds were released in similar amounts by the frass from both the species fed either type of food (maize leaves versus the artificial diet) (Table 1). Among those compounds, ethyl palmitate elicited a strong EAG response in *M. mediator* and attracted the parasitoids.

Palmitic acid and ethyl palmitate are possibly common by-products of insect digestion—Palmitic acid is one of the most abundant saturated fatty acids in many biological organisms, including plants [65]. The compound or its derivatives (e.g., ethyl palmitate) are sometimes found in frass (or other anal secretions) of herbivorous insects, including stink bugs, fruit flies, and beetles [66,67,68]. Our data showed that palmitic acid and ethyl palmitate also occurred in larval frass of the noctuid species. As mentioned above and shown in Table 1, the proportions (or amounts) of these compounds remained relatively similar across the frass from the larvae fed the two types of food, possibly because they were by-products of larval digestion of plant tissues.

In addition, gut bacteria that have been believed to be essential in digestion in insects [69] also contributed to greater weight gains in the two noctuid larvae, which was apparent after a relatively long period of feeding (6 days), but generally not statistically significant after a relatively short period of feeding (3 days). This was possibly because the gut bacteria completely lost their function only after several days of the antibiotic treatment. However, we observed that the survival rates were not reduced after 6 days of feeding on the tetracycline-treated diet.

How might VOCs of gut symbionts mediate predator–prey interactions?—Microbes are talented chemists in producing large amounts of VOCs with high compound diversity, diverse metabolic pathways and multiple ecological roles [70]. When they become insect symbionts, their VOCs are expected to imbue host insects with exceptional smells that have multiple functions in interactions with their natural enemies. For example, mandelonitriles released by gut bacteria of the beetle *L. decemlineata* are used by the insect hosts to defense against natural enemies [35]. In contrast, VOCs of gut bacteria often betray hosts by attracting natural enemies [39,40,71], as also found in this study. Two stereoisomers of 2,3-butanediol are the typical VOCs of bacteria [59]. In our study, we found that they also occurred in the larval frass of both the noctuid species. Interestingly, supplementing their diets with tetracycline completely ceased the release of the compounds from the larvae frass in both the moth species. Meanwhile, the amounts of the attractant ethyl palmitate were also reduced. These findings introduce the idea that gut digestion, involving bacteria, contributes to the formation of VOCs of frass, which shape important predator–prey interactions, as found here and in several other studies [35,37].

## 5. Conclusions

The chemical compositions of the noctuid larval bodies and frass remained relatively similar, which were also largely independent of the diet types. Larval frass, rather than the bodies of both the species, was significantly attractive to the larval endoparasitoid *M. mediator.* A ubiquitous compound, ethyl palmitate, found in the frass of both the species was responsible for the attraction of *M. mediator* and was likely to be one of common metabolites of gut digestion involving symbionts. This study contributes to a better understanding of host–parasitoid interactions, which might be important for the developments of biological control programs in the future, for example, by using ethyl palmitate as an attractant to natural enemies for enhancing biological control services on crops.

## Figures and Tables

**Table 1:** Amounts (ng) of compounds in larval frass or bodies of the two noctuid species when they were fed either maize leaves or the artificial diet for 6 days.

Compounds	*Ha* Frass		*Sf* Frass		*Ha* Larvae		*Sf* Larvae	
	Maize	AD	Maize	AD	Maize	AD	Maize	AD
**2,3-butanediol I ^▲^**	63 ± 16 a	95 ± 13 a	23 ± 7 a’	11 ± 4 a’	n.d.	n.d.	n.d.	n.d.
**2,3-butanediol II ^▲^**	42 ± 12 a	54 ± 17 a	27 ± 9 a’	21 ± 8 a’	n.d.	n.d.	n.d.	n.d.
**Benzaldehyde ^◇^**	13 ± 2	n.d.	11 ± 3	n.d.	n.d.	n.d.	n.d.	n.d.
**2-ethylhexanol ^◇^**	10 ± 2	n.d.	14 ± 5	n.d.	n.d.	n.d.	n.d.	n.d.
**(*E*)-coniferol ^◇^**	7 ± 1	n.d.	16 ± 5	n.d.	n.d.	n.d.	n.d.	n.d.
**Pentadecane ^B^**	17 ± 6 a	19 ± 7 a	16 ± 4 a’	13 ± 3 a’	**183 ± 21 a’’**	**156 ± 28 a’’**	**245 ± 62 a’’’**	**215 ± 31 a’’’**
**palmitoleic acid**	47 ± 10 a	41 ± 13 a	24 ± 10 a’	26 ± 6 a’	n.d.	n.d.	n.d.	n.d.
**palmitic acid ^F^**	**271 ± 51 a**	**254 ± 34 a**	**286 ± 45 a’**	**213 ± 31 a’**	21 ± 6 a’’	33 ± 9 a’’	18 ± 6 a’’’	9 ± 3 a’’’
**ethyl palmitate**	81 ± 12 a	73 ± 11 a	93 ±17 a’	74 ± 22 a’	n.d.	n.d.	n.d.	n.d.

Pairwise comparisons were conducted with the *t*-test to analyze the differences in the amounts of compounds of the larval frass when larvae of *Helicoverpa armigera* (abbr. as *Ha*) and *Spodoptera frugiperda* (abbr. as *Sf*) were fed either maize leaves or the artificial diet. Similar analyses were likewise performed on the data of the larval bodies. As a result, the amounts of two 2,3-butanediol stereoisomers, pentadecane, palmitoleic acid, palmitic acid, and ethyl palmitate were independent of types of food in the frass or body extracts of both the species (*p* > 0.05, *n* = 5, indicated by the parallel symbols of a, a’, a’’, and a’’’ in cells). However, three compounds (indicated by “◇” in cells) were found in the frass of both the species when their larvae were fed maize leaves, but were not detected (abbr. as n.d. in cells) in the frass of those fed the artificial diet or in the body samples. The superscript letter “B” indicates that the larval bodies released significant higher amounts (bold numbers) of the compounds than did their respective larval frass, regardless of the diet types. On the contrary, “F” indicates that the larval frass included higher amounts (bold numbers) of compounds than did the corresponding bodies (i.e., pairwise comparisons between the body and frass samples of either species fed one of the two diets, *t*-test, *p* < 0.05, *n* = 5). (▲) 2,3-butanediol I was identified as (2*S*,3*S*)-(+)-butanediol, and 2,3-butanediol II was meso (*R*,*S*)-2,3-butanediol.

## Data Availability

All data used in this manuscript were archived on Mendeley Data at https://data.mendeley.com/drafts/wyrsb3py54 (accessed on 6 July 2025) (DOI: 10.17632/wyrsb3py54.1).
